# Integral Analyses of Competing Endogenous RNA Mechanisms and DNA Methylation Reveal Regulatory Mechanisms in Osteosarcoma

**DOI:** 10.3389/fcell.2021.763347

**Published:** 2021-12-09

**Authors:** Tingrui Wu, Bo Wei, Hao Lin, Boan Zhou, Tao Lin, Qianzheng Liu, Hongxun Sang, Huan Liu, Wenhua Huang

**Affiliations:** ^1^ Orthopaedic Center, Affiliated Hospital of Guangdong Medical University, Zhanjiang, China; ^2^ Department of Orthopaedics, Shenzhen Hospital, Southern Medical University, Shenzhen, China; ^3^ Department of Orthopaedics, Affiliated Traditional Chinese Medicine Hospital, Southwest Medical University, Luzhou, China; ^4^ National Traditional Chinese Medicine Clinical Research Base, The Affiliated Traditional Chinese Medicine Hospital of Southwest Medical University, Luzhou, China; ^5^ Guangdong Provincial Key Laboratory of Medical Biomechanics, National Key Discipline of Human Anatomy, Guangdong Engineering Research Center for Translation of Medical 3D Printing Application, School of Basic Medical Sciences, Southern Medical University, Guangzhou, China; ^6^ Guangdong Medical Innovation Platform for Translation of 3D Printing Application, The Third Affiliated Hospital of Southern Medical University, Guangzhou, China

**Keywords:** bioinformatics, osteosarcoma, competing endogenous RNA, circRNA, methylation

## Abstract

**Background:** Osteosarcoma (OS) is the most common primary malignant bone tumour in children and adolescents, with rapid growth, frequent metastasis, and a poor prognosis, but its pathogenesis has not been fully elucidated. Exploring the pathogenesis of OS is of great significance for improving diagnoses and finding new therapeutic targets.

**Methods:** Differentially expressed circRNAs (DECs), miRNAs (DEMs), methylated DNA sites (DMSs), and mRNAs (DEGs) were identified between OS and control cell lines. GSEA of DEGs and functional enrichment analysis of methylated DEGs were carried out to further identify potential biological processes. Online tools were used to predict the miRNA binding sites of DECs and the mRNA binding sites of DEMs, and then construct a circRNA-miRNA-mRNA network. Next, an analysis of the interaction between methylated DEGs was performed with a protein-protein interaction (PPI) network, and hub gene identification and survival analysis were carried out. The expression pattern of circRNA-miRNA-mRNA was validated by real-time PCR.

**Results:** GSEA and functional enrichment analysis indicated that DEGs and methylated DEGs are involved in important biological processes in cancer. Hsa_circ_0001753/has_miR_760/CD74 network was constructed and validated in cell lines. Low expression levels of CD74 are associated with poor overall survival times and show good diagnostic ability.

**Conclusion:** Methylated DEGs may be involved in the development of OS, and the hsa_circ_0001753/has_miR_760/CD74 network may serve as a target for the early diagnosis of and targeted therapy for OS.

## Introduction

Osteosarcoma (OS) is the most common malignant bone tumour and occurs primarily in the metaphysis of long bones in children and adolescents (Berner et al., 2015). The annual incidence of OS ranges from 1 to 4 in 1 million and is slightly higher in men than in women (Mirabello et al., 2009). Although the overall incidence of OS is low, its prognosis is poor. With the combined application of limb salvage surgery and neoadjuvant chemotherapy, the 5-year survival rate for patients without metastases is approximately 65–70% (Siegel et al., 20182018). Approximately 15–20% of patients have evidence of metastasis at the time of diagnosis, mainly to the lung, and the prognosis for patients with metastatic disease remains poor (Meazza and Scanagatta, 2016). Many OS patients are insensitive to chemotherapy drugs or have developed resistance, and the recurrence and widespread metastasis of tumours also complicate the clinical treatment of osteosarcoma. To date, although molecular biological research related to osteosarcoma has provided a theoretical basis for its pathogenesis, the molecular mechanisms of the malignant biological behaviour of osteosarcoma is still unclear.

With the development of a new generation of sequencing technology, noncoding RNA (ncRNA) has gradually become a hot topic of research regarding tumour pathogenesis. circRNAs are ncRNAs that play a role in biological regulation, primarily through genetic regulation (Li et al., 2018). circRNA is a single-stranded endogenous noncoding RNA produced by reverse splicing; it has a covalent closed-loop structure that cannot be easily degraded by RNA nucleic acid exonuclease (Chen and Yang, 2015). circRNAs have abundant miRNA binding sites and can absorb miRNAs as sponges (Tay et al., 2014). circRNAs can compete with miRNAs to affect the stability of target RNAs or their translation, thus regulating gene expression at the post-transcriptional level (Zhong et al., 2018). At the same time, circRNAs are widely involved in the processes of cell proliferation, apoptosis, invasion and migration, playing an important regulatory role in human diseases (Wang et al., 2020a; Li et al., 2020; Yu et al., 2020). Based on competing endogenous RNA (ceRNA) mechanisms, a variety of abnormally expressed circRNAs play an important regulatory role in cancer, including breast cancer (Sang et al., 2019; Yang et al., 2019), lung cancer (Cheng et al., 2019; Chen et al., 2020), liver cancer (Yu et al., 2019; He et al., 2020) and gastric cancer (Zhang et al., 2019; Luo et al., 2020). As a ceRNA, circ-0001785 mediates miR-1200 to regulate the pathogenesis of OS by acting as a molecular sponge, thereby upregulating HOXB2 and mediating the malignant cell behaviour of OS cells (Li et al., 2019). Another study revealed that the high expression of circ-03955 promotes EMT in osteosarcoma by acting as a miR-3662 sponge-mediated MTDH expression (Wang et al., 2020b). However, many biological functions of circRNAs in osteosarcoma are still unknown.

Many studies in recent years have shown that epigenetic changes play an important role in regulating the occurrence and development of tumours. DNA methylation is one of the most common epigenetic events involving the predominantly reversible addition of methyl groups to cytosines without altering the genomic DNA sequence in the context of CpG dinucleotides (Ding et al., 2019). Studies have shown that the promoter regions of tumour suppressor genes are hypermethylated and inhibited, while oncogenes are hypomethylated and abnormally active (Lin and Wang, 2014). Abnormal DNA methylation has been proven to regulate tumorigenesis and the progression of various cancers (Skvortsova et al., 2019; Wang et al., 2020c). Many studies have reported findings related to DNA methylation in OS. According to reports, overexpression of SENP3 can increase DNA methylation of E-Cad and then increase the proliferation, migration and invasion of osteosarcoma cells (Yang et al., 2020). The high expression level of WNT6 in primary osteosarcoma is mainly due to the low DNA methylation level of WNT6, while the DNA methylation of WNT6 shows a negative correlation with the prognosis of children with osteosarcoma (Li et al., 2017). Although the DNA methylation pattern in OS has attracted attention as an important biomarker and therapeutic target, the mechanism by which ceRNA and DNA methylation regulate OS is still not understood.

**FIGURE 1:**
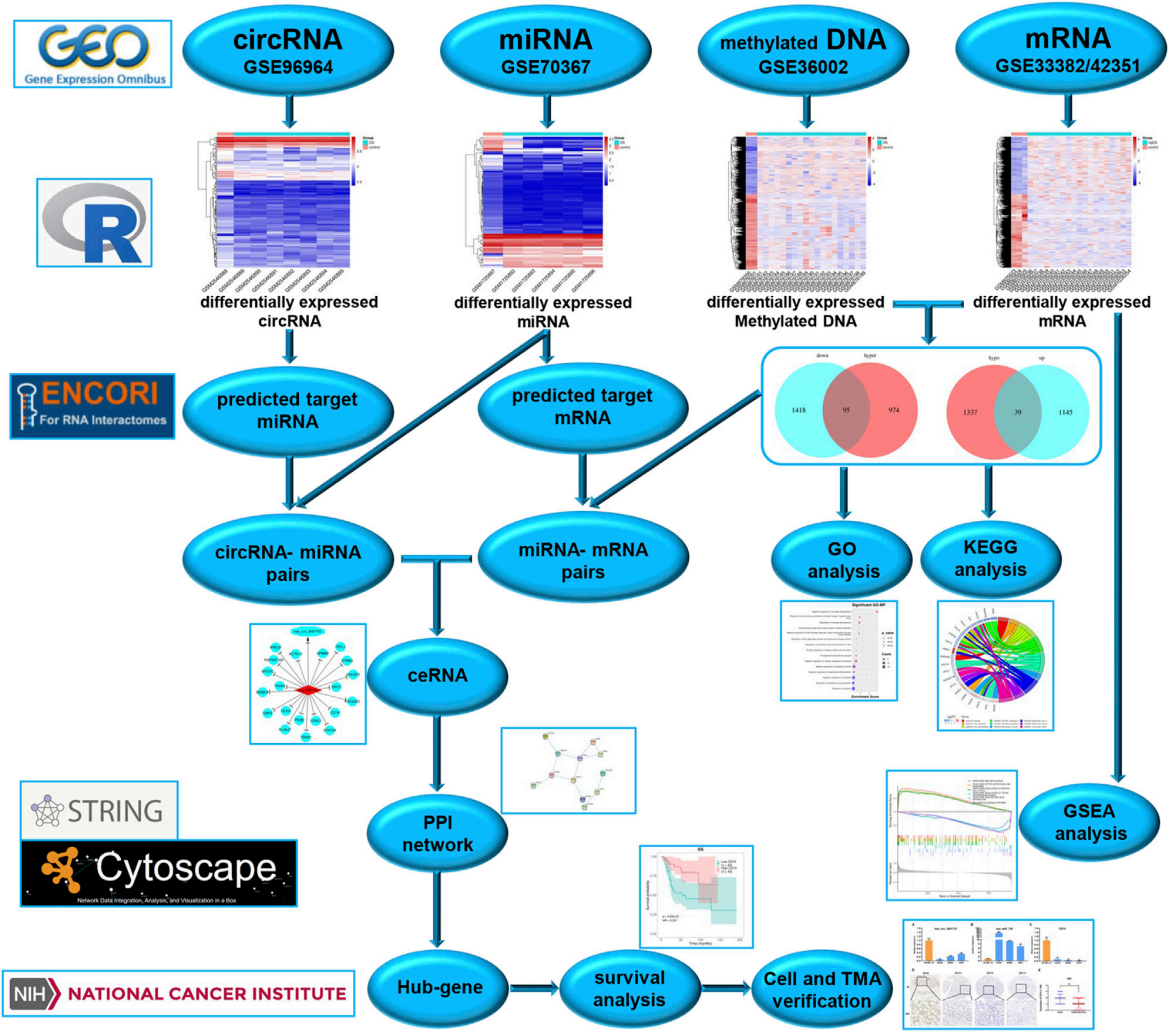
Study flow chart.

To better understand the molecular mechanisms of OS, we used gene microarray and bioinformatics analysis to study the potential mechanism of ceRNA and DNA methylation in OS. The flow chart summarizing this work is shown in Figure 1. In this study, OS-related datasets were selected from the gene microarray of the GEO database, and differentially expressed circRNAs (DECs), miRNAs (DEMs), methylated DNA sites (DMSs) and mRNAs (DEGs) were identified between OS and control cell lines. Functional enrichment analysis of DEGs and GSEA of methylated DEGs were carried out to further identify potential biological processes. On the basis of the ceRNA mechanism, online tools were used to predict the miRNA binding sites of DECs and the mRNA binding sites of DEMs and then construct a circRNA-miRNA-mRNA network. Next, a protein-protein interaction (PPI) network was established to analyse interactions between methylated DEGs, and then hub gene identification and survival analysis were carried out. Finally, the ceRNA mechanism of the circRNA-miRNA-mRNA network was verified in cell lines and tissues. The aims of our research were to identify and verify the ceRNA mechanism and methylated DEGs in OS. Our findings provide new insights into the potential carcinogenic effects of abnormal DNA methylation and identify potential biomarkers and therapeutic targets of OS.

## Materials and Methods

### Microarray Datasets

The Gene Expression Omnibus (GEO; http://www.ncbi.nlm.nih.gov/geo) is a public platform for storing genetic data. One circRNA expression profiling dataset (GSE96964 [platform: GPL19978 Agilent-69978 Arraystar Human CircRNA microarray V1]), one miRNA expression profiling dataset (GSE70367 [platform: GPL16384 Affymetrix Multispecies miRNA-3 Array]), one DNA methylation expression profiling dataset (GSE36002 [platform: GPL8490 Illumina HumanMethylation27 BeadChip]), and two gene expression profiling datasets (GSE42351 and GSE33382 [platform: Illumina human-6 v2.0 expression BeadChip]) were downloaded from the GEO database. The GSE96964 dataset includes 7 human osteosarcoma cell lines (OS group) and 1 human osteoblast (control group); GSE70367 includes 5 human osteosarcoma cell lines (OS group) and 1 human mesenchymal stem cell line (control group); GSE36002 includes 19 human osteosarcoma cell lines (OS group) and 2 human osteoblasts (control group); GSE42351 includes 19 human osteosarcoma cell lines (OS group) and GSE33382 includes 3 human osteoblasts (control group).

### Intragroup data Repeatability Test

Principal component analysis (PCA) is a general method of sample clustering analysis, which is often used for sample clustering based on various variable information, such as gene expression, resequencing, and diversity analysis. The intragroup data repeatability verification of the dataset was tested by PCA. Pearson’s correlation test was used to verify the repeatability of the data in each group. The statistical analysis and visual heatmap were drawn via the R programming language and depict the correlations among intragroup data.

### Microarray Analysis

The limma package (Ritchie et al., 2015) in R was used to identify differentially expressed circRNAs (DECs), miRNAs (DEMs), methylated DNA sites (DMSs) and mRNAs (DEGs) between OS and control cell lines. |Log2FC| ≥ 0.263, *P* value ＜ 0.05 and q value ＜ 1 were considered statistically significant differences. For the selected DECs, DEMs, DMSs and DEGs, we generated volcano plots and heatmaps using R software.

### Gene Set Enrichment Analysis

According to the differentially expressed genes, GSEA (Subramanian et al., 2005) was used for gene collection enrichment analysis. Genome enrichment analysis is a computational method used to determine whether a given gene set differs significantly between different groups. The genes in these clusters are related in some way, such as having the same biological function, being located close to each other on the chromosome, or being self-defined according to certain criteria. Therefore, gene set enrichment analysis can compensate for the deficiencies of single-gene analysis.

### GO and KEGG Analysis of Methylated DEGs

Gene Ontology (GO) analysis is widely used for annotating genes and determining biological characteristics, including three key biological aspects: cellular components (CC), molecular function (MF), and biological process (BP) (Ashburner et al., 2000). The Kyoto Encyclopedia of Genes and Genomes (KEGG) pathway database is one of the most commonly applied databases for systematically analysing gene functions (Kanehisa and Goto, 2000). The Database for Annotation, Visualization and Integrated Discovery (DAVID, https://david.ncifcrf.gov/) is a gene functional classification tool designed to provide authors with a comprehensive set of functional annotation tools to understand the biological significance behind a large number of genes, and which can be used to perform GO and KEGG analysis. A *P* value < 0.05 was considered statistically significant.

### CeRNA Network Construction

In this study, we used the starBase database (Li et al., 2014) (http://starbase.sysu.edu.cn/) to predict the miRNA binding sites of DECs. Based on DEMs, we further screened target mRNAs. Then, the interactions between miRNAs and mRNAs were predicted by the miRanda (John et al., 2004), miRDB (Wong and Wang, 2015), miTarBase (Hsu et al., 2011), TargetScan (Agarwal et al., 2015) and miRMap (Vejnar et al., 2013) databases. The Cancer-Specific CircRNA Database (http://gb.whu.edu.cn/CSCD/) was used to understand the structural model of circRNAs, including miRNA binding sites (MREs), RNA binding proteins (RBPs), and open reading frames (ORFs). According to the circRNA-miRNA and mRNA-miRNA interactions, Cytoscape software 3.7 (Shannon et al., 2003) (https://cytoscape.org/) was used to visualize the ceRNA network of circRNA-miRNA-mRNA.

### PPI Network Construction and Hub Gene Identification

For genes in the ceRNA network, the Search Tool for the Retrieval of Interacting Genes (STRING) (Mering et al., 2003) (https://string-db.org/) online tool was used to analyse protein-protein interactions (PPIs). The required confidence (combined score) > 0.15 was selected as the threshold for protein-protein interactions. Based on the obtained PPI relationship pairs, Cytoscape was used to analyse the topology of the PPI relationship network. From the obtained biological networks, we observed that most biological networks conformed to the attributes of a scale-free network. Therefore, the primary node involved in the protein interaction relationship in the PPI network, namely, the hub gene (He and Zhang, 2006), could be obtained by using connectivity degree analysis in network statistics. In this study, the nodes of the network were analysed, and the hub genes were identified with degree > 2.

### Survival Analysis of the Hub Genes

The study attempted to validate the hub genes from the TCGA datasets of OS. The expression level of each hub gene was extracted for further analysis from all included data. Survival analysis and ROC curve analysis were performed by using the Survival Roc package according to the OS expression values of key genes in TCGA datasets. Survival analysis was carried out through Kaplan-Meier analysis, and the log-rank test was performed to evaluate the statistical significance of differences. The significance cut-off was fixed at a p value < 0.05.

### Cell Culture

Human osteoblasts (hFOB1.19) and OS cell lines (U2OS, MG63, HOS) were cultured according to a previously reported protocol (Lin et al., 2020).

### RNA Isolation and RT-PCR

Total RNA was isolated from cultured cells using TRIzol reagent (Invitrogen, Carlsbad, CA, United States) according to the manufacturer’s instructions. The extracted RNA was quantified by spectrophotometry using NanoDrop equipment (Thermo Fisher Scientific, Waltham, MA, United States). Reverse transcription of mRNA and miRNA was conducted using random primers and tail primers in the TaKaRa system (Dalian, China), respectively.

Real-time PCR was performed using TB Green PCR Master Mix (Takara) with an ABI StepOne Real-Time PCR System (Applied Biosystems 7500, Foster City, CA, United States). The expression levels of circRNA and mRNA were normalized according to GAPDH expression, and miRNA was normalized according to U6 expression by using an optimized comparative Ct (2−ΔΔCt) value method. Real-time PCRs were performed in triplicate.

The primers used were as follows: hsa_circ_0001753 (forward, 5′-AGG​ACT​CGT​TCC​GTC​TGT​CAC​TC-3′ and reverse, 5′-AAT​GGT​CTG​GCA​CGA​GGA​ATC​AAC-3′), hsa_miR_760 (CGG​CTC​TGG​GTC​TGT​GG), CD74 (forward, 5′-CTT​TTC​CAT​CCT​GGT​GAC​TCT-3′ and reverse, 5′-GAG​GTG​ACT​GTC​AGT​TTG​TCC-3′), GAPDH (forward, 5′-CAC​CCA​CTC​CTC​CAC​CTT​TG-3′ and reverse, 5′-CCA​CCA​CCC​TGT​TGC​TGT​AG-3′) and U6 (forward, 5′-TGG​AAC​GCT​TCA​CGA​ATT​TGC​G-3′ and reverse, 5′-GGA​ACG​ATA​CAG​AGA​AGA​TTA​GC-3′). The size of the PCR product: hsa_circ_0001753: 112 bp/hsa_miR_760: 60 bp/CD74: 99 bp/GAPDH: 138 bp/U6: 68 bp.

### Tissue Microarray and Immunohistochemistry

Paraffin-embedded TMAs used in the present study were purchased from Bioaitech (Bioaitech, Xian, China). Osteosarcoma and normal bone tissue TMAs contain 70 cancer samples and 20 normal bone tissue samples. IHC was utilized to determine the relative expression of CD74 in 90 samples by means of the manufacturer’s instructions. Primary antibodies against CD74 (1:500, Cell Signaling Technology, United States) were used. HRP-labeled streptavidin solution was added into the samples for 15 min after both primary and secondary antibody incubations. The immunocomplex was visualized with DAB, and the nucleus were counterstained with haematoxylin. Pictures were taken with a biochip scanner (Pannoramic MIDI, 3D HISTECH, Hungary). The expressions of CD74 were quantified and analyzed. The staining was scored according to the previously described 4-point system (score 0–3) as follows: score 3, dark staining that is easily visible and present in >50% of cells; score 2, focal areas of dark staining (<50% of cells) or moderate staining of >50% of cells; score 1, focal moderate staining in <50% of cells or pale staining in any proportion of cells not easily observable at low power; and score 0, none of the aforementioned (Deng et al., 2019). A high level of expression was defined as a score of 2–3 and low level of expression was defined as a score of 0–1, as described previously.

### Bisulfite Sequencing PCR

Genomic DNA from osteoblasts and osteosarcoma cells was isolated using the Ezup Column Animal Genomic DNA Purification Kit (Sangon Biotech, B518251) according to the manufacturer’s instructions. By treatment of genomic DNA with sodium bisulfite, all unmethylated cytosine (C) is converted to uracil (U) while methylated cytosine remains unchanged. After the sulfite treatment of genomic DNA, BSP primers were designed to amplify the target fragments, at which time uracil (U) was all converted into thymine (T). Finally, the PCR products were sequenced to determine whether methylation of CpG sites occurred. The CpG island of 2000 bp sequence before exon 1 of CD74 gene was predicted. The primers used to amplify the bisulfite-treated DNA were designed using MethPrimer software. The BSP primers were as follows: F: 5′-TGT​TGT​TTA​GTT​AGT​GTA​GG-3'; R: 5′- AAAAAAACCAAACACRATAACTCA-3'. The PCR products were purified and cloned, and 10 clones from each group of samples were selected for sequencing, and sequencing was done by Sangon Biotech.

### Statistical Analysis

Statistical analyses were conducted by GraphPad Prism 7.0 (GraphPad Software Inc., CA, United States). Data are expressed as the mean ± standard deviation (SD) and were assessed using unpaired Student’s t-tests.

## Results

### Validation of the Datasets

**FIGURE 2:**
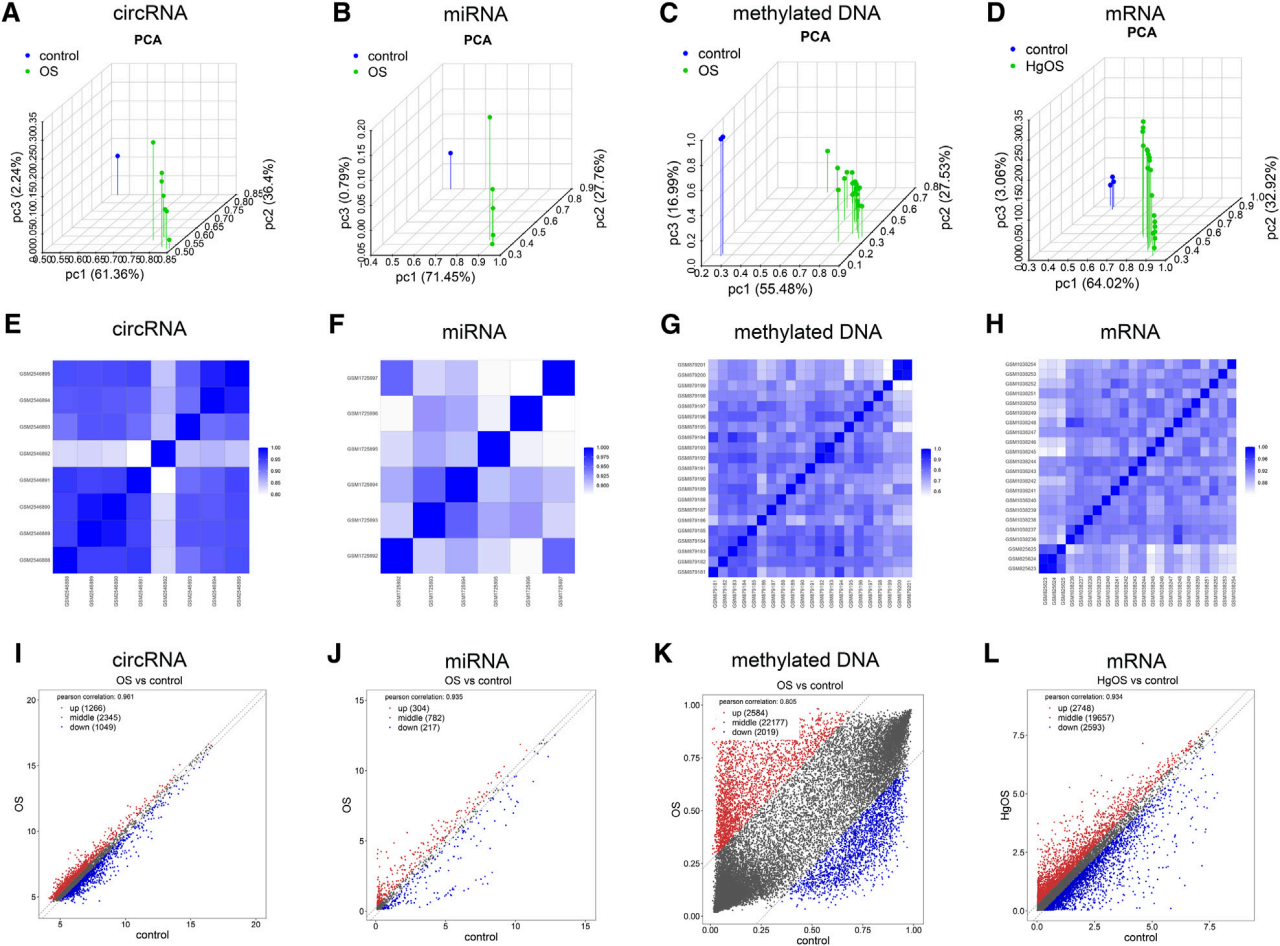
Intragroup data repeatability test for the circRNA miRNA, methylated DNA and mRNA datasets through PCA and Pearson’s correlation analysis. **(A–D)** PCA of samples from the circRNA, miRNA, methylated DNA and mRNA datasets. The scatter plots are plotted on the x-, y-, and z-axes for PC1, PC2, and PC3, respectively, with each point representing a sample. The further the distance between the two samples was, the larger the difference in gene expression profiles of the two samples would be. **(E–H)** Pearson’s correlation analysis of the circRNA, miRNA, methylated DNA and mRNA datasets. The colour of heatmaps reflects the intensity of correlation. When 0 < correlation < 1, there is a positive correlation. When −1 < correlation < 0, there is a negative correlation. The greater the absolute value of numbers, the stronger the correlation. **(I–L)** Scatter diagrams showed Pearson’s correlation. PC1, principal component 1; PC2, principal component 2; PC3, principal component 3; PCA, principal component analysis.

To further validate the repeatability of intragroup data, PCA and Pearson’s correlation test were used. Based on the PCA, the intragroup data repeatability for the circRNA, miRNA, methylated DNA and mRNA datasets was acceptable. In all four datasets, the distance between the intragroup samples in the control and OS groups was close, and the distance between the control and case groups was considerable (Figure 2A–D). Based on Pearson’s correlation test, it was found that in the circRNA, miRNA, methylated DNA and mRNA datasets, there was a strong correlation within the control group samples, and there was also a strong correlation within the OS group samples. The heatmap (Figure 2E–H) and scatter diagram (Figure 2I–L) depict Pearson’s correlation.

### DECs, DEMs, DMSs, DMGs, and DEGs

**FIGURE 3:**
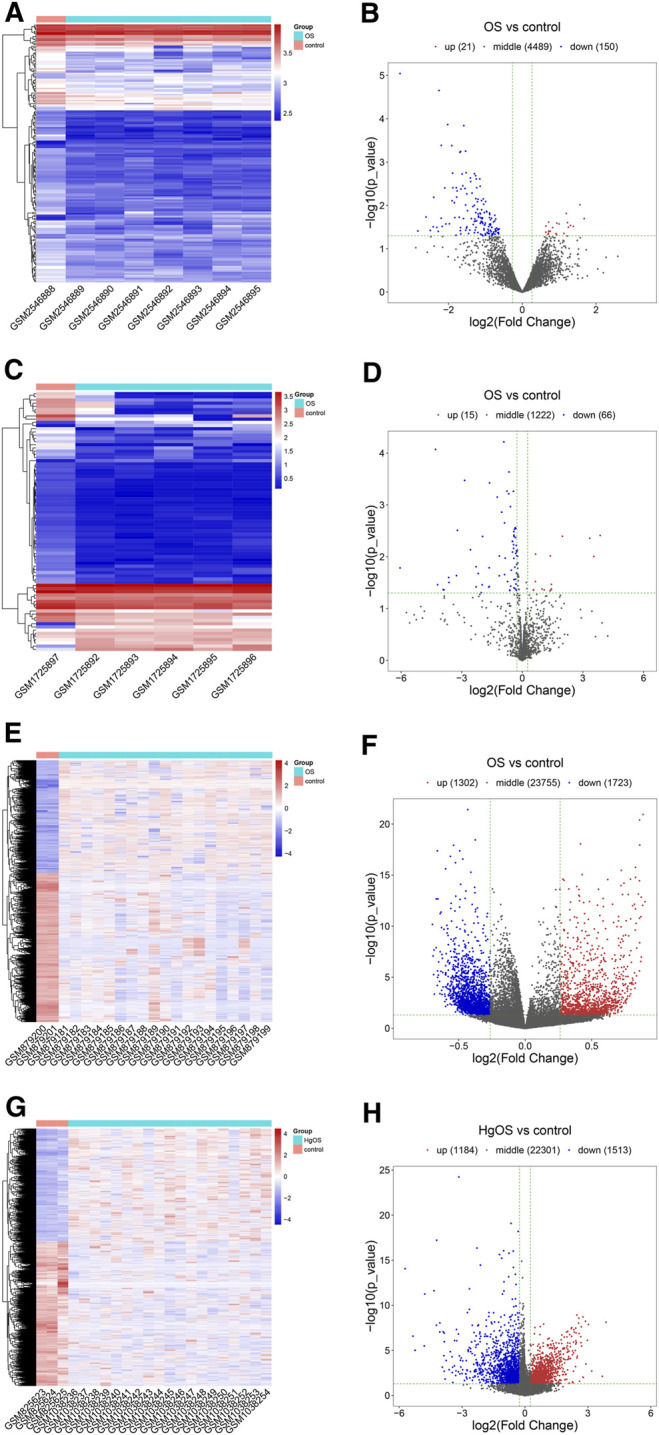
Heatmaps and volcano plots of DECs, DEMs, DMSs and DEGs in OS. In the hierarchical cluster heatmap, red and blue indicate high and low expression, respectively, with columns representing samples and rows representing, respective RNAs or methylated sites. Volcano plots were used to distinguish the differentially expressed RNAs or methylated sites. The vertical lines correspond to a difference of 0.263-fold, and the horizontal line represents a P value of 0.05. **(A,B)** Twenty-one upregulated and 150 downregulated circRNAs were identified from differentially expressed circRNAs; **(C,D)** 15 upregulated and 66 downregulated miRNAs were identified from differentially expressed miRNAs; **(E,F)** 3,025 methylated DNA sites were identified from differentially methylated DNA sites; **(G,H)** 1,184 upregulated and 1,513 downregulated mRNAs were identified from differentially expressed mRNAs.

After expression profile processing, there were a total of 4,660 circRNAs, 1,303 miRNAs, 26,780 methylated DNA sites and 24998 mRNAs in the expression profiles used for identifying differential expression. According to the cut-off criteria of |Log2FC| ≥ 0.263, p value ＜ 0.05 and q value ＜ 1, a total of 171 differentially expressed circRNAs (DECs, 21 up- and 150 downregulated); 81 differentially miRNAs (DEMs, 15 up- and 66 downregulated); 3,025 differentially methylated DNA sites (DMSs), covering 2,445 differentially methylated genes (DMGs, 1,069 hypermethylated and 1,376 hypomethylated) and 2,697 differentially expressed mRNAs (DEGs, 1,184 up- and 1,513 downregulated) were identified. The detailed results of differential expression are displayed in hierarchical cluster heatmaps and volcano plots (Figure 3).

### GSEA

**FIGURE 4:**
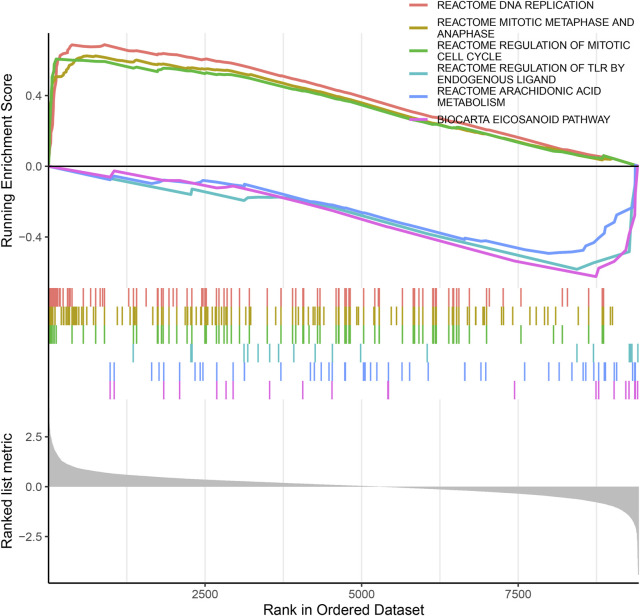
The GSEA of DEGs datasets. The top portion of plots shows the enrichment scores for each gene, and the bottom portion shows the ranked genes. Y-axis: ranking metric, X-axis: individual ranks for all genes.

To identify the biological functional mechanism in OS, the expression matrix constructed from DEG analysis was used for GSEA based on the C2 database. GSEA revealed that the most significantly enriched gene set in OS was the REACTOME DNA REPLICATION, and other significant gene sets included REACTOME MITOTIC METAPHASE AND ANAPHASE, REACTOME REGULATION OF MITOTIC CELL CYCLE, REACTOME REGULATION OF TLR BY ENDOGENOUS LIGAND, REACTOME ARACHIDONIC ACID METABOLISM and BIOCARTA EICOSANOID PATHWAY (Figure 4). These enriched pathways revealed that DEGs were closely associated with the malignant characteristics of OS, especially apoptosis and the cell cycle.

### Screening for Methylated DEGs, GO and KEGG Pathway Analysis

**FIGURE 5:**
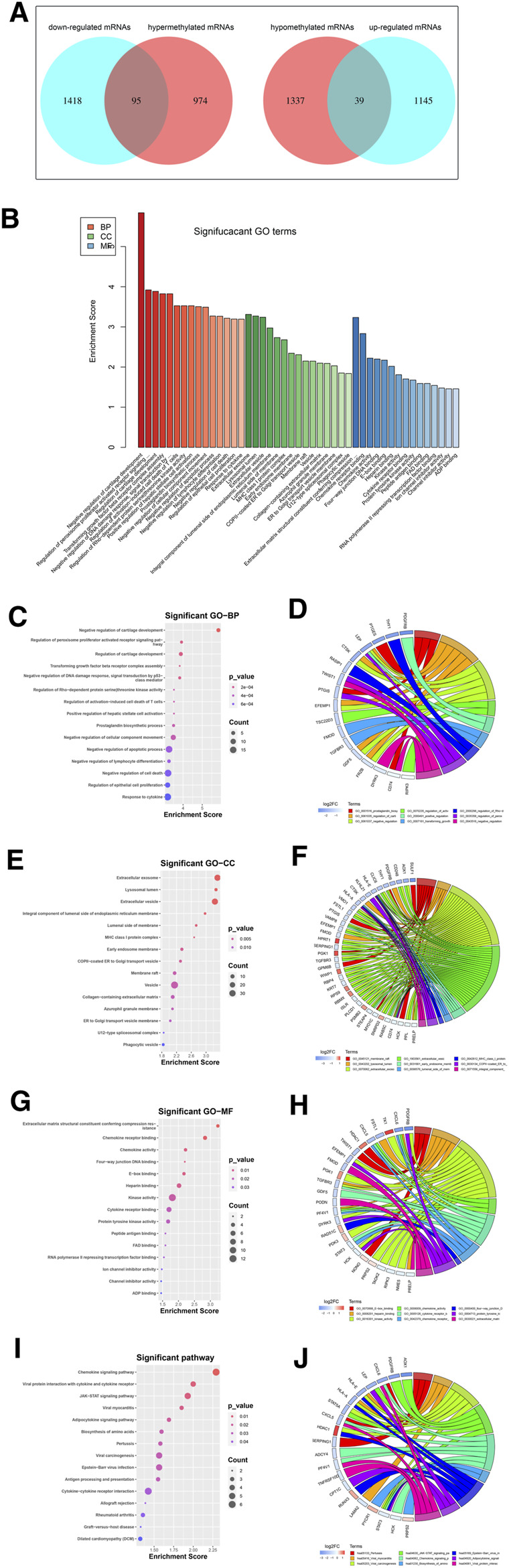
Identification and enrichment analysis of methylated DEGs. **(A)** A total of 95 hypermethylated DEGs and 39 hypomethylated DEGs were screened by Venn diagram; **(B)** GO analysis including CC, MF, and BP biological aspects; **(C)** BP: The top 15 enriched GO terms of the methylated DEGs; **(D)** BP: The methylated DEGs of the top 9 enriched GO terms; **(E)** CC: The top 15 enriched GO terms of the methylated DEGs; **(F)** CC: The methylated DEGs of the top 9 enriched GO terms; **(G)** MF: The top 15 enriched GO terms of the methylated DEGs; **(H)** MF: The methylated DEGs of the top 9 enriched GO terms; **(I)** KEGG: The top 15 enriched Reactome pathways of the methylated DEGs; **(J)** KEGG: The methylated DEGs of the top 9 enriched Reactome pathways.

Among the DEGs, a total of 95 hypermethylated downregulated mRNAs (hypermethylated DEGs) were screened from 1,069 overlapping hypermethylated mRNAs and 1,513 downregulated mRNAs, while 39 hypomethylated upregulated mRNAs (hypomethylated DEGs) were screened from 1,376 overlapping hypomethylated mRNAs and 1,184 upregulated mRNAs. The 134 methylated DEGs are presented in a Venn diagram (Figure 5A).

To further understand the gene function, we performed 134 methylated DEG enrichment functions determined by the DAVID online analysis tool, and the GO analysis of DEGs was mainly classified into three functional groups: BP, CC, and MF (Figure 5B). In the BP group, the GO term analysis indicated that the methylated DEGs were primarily enriched in functions such as negative regulation of cartilage development, regulation of the peroxisome proliferator activated receptor signalling pathway, regulation of cartilage development, transforming growth factor beta receptor complex assembly, negative regulation of the DNA damage response and signal transduction by the p53 class mediator (Figures 5C,D). The top 15 enriched GO-BP terms of the methylated DEGs are listed in Table 1. In the CC group, the results of GO analysis were enriched in extracellular exosomes, lysosomal lumen, extracellular vesicles, integral components of the luminal side of the endoplasmic reticulum membrane, and the luminal side of membranes (Figures 5E,F). The top 15 enriched GO-CC terms of the methylated DEGs are listed in Table 2. In the MF group, the enriched GO terms were primarily involved in extracellular matrix structural constituents conferring compression resistance, chemokine receptor binding, chemokine activity, four-way junction DNA binding and E-box binding (Figures 5G,H). The top 15 enriched GO-MF terms of the methylated DEGs are listed in Table 3.

**TABLE 1:** The top 15 enriched GO-BP terms of the methylated DEGs.

ID	Term	Count	p value	Fdr	Enrichment score	Genes
GO:0061037	negative regulation of cartilage development	5	1.43811E-06	0.00329472	5.842206525	CTSK//EFEMP1//FRZB//GDF5//LEP
GO:0035358	regulation of peroxisome proliferator activated receptor signaling pathway	3	0.000120294	0.06854149	3.919755243	LEP//PTGIS//TWIST1
GO:0061035	regulation of cartilage development	5	0.000130348	0.06854149	3.884894	CTSK//EFEMP1//FRZB//GDF5//LEP
GO:0007181	transforming growth factor beta receptor complex assembly	2	0.000149499	0.06854149	3.825361853	FMOD//TGFBR3
GO:0043518	negative regulation of DNA damage response, signal transduction by p53 class mediator	3	0.000149589	0.06854149	3.825101543	CD74//DYRK3//TWIST1
GO:0070235	regulation of activation-induced cell death of T cells	2	0.000297605	0.073879265	3.526359232	RIPK3//TSC22D3
GO:2000298	regulation of Rhodependent protein serine|threonine kinase activity	2	0.000297605	0.073879265	3.526359232	RASIP1//THY1
GO:2000491	positive regulation of hepatic stellate cell activation	2	0.000297605	0.073879265	3.526359232	LEP//PDGFRB
GO:0001516	prostaglandin biosynthetic process	3	0.000312027	0.073879265	3.505807423	CD74//PTGES//PTGIS
GO:0051271	negative regulation of cellular component movement	9	0.000322476	0.073879265	3.491502524	CD74//CYGB//PODN//SEMA3F//SPINT2//STAT3//SULF1//TGFBR3//THY1
GO:0043066	negative regulation of apoptotic process	16	0.000536326	0.091473127	3.270571437	CD74//DYRK3//GDF5//HCK//HDAC1//HSPB6//LEP//LIMS2//NME5//NONO//PDGFRB//PRAME//STAT3//TNFRSF10D//TSC22D3//TWIST1
GO:0045620	negative regulation of lymphocyte differentiation	4	0.000542831	0.091473127	3.26533551	CD74//HMGB3//LOXL3//RUNX3
GO:0060548	negative regulation of cell death	17	0.000608971	0.091473127	3.21540335	CD74//DYRK3//GDF5//HCK//HDAC1//HSPB6//LEP//LIMS2//NME5//NONO//PDGFRB//PRAME//PYCR1//STAT3//TNFRSF10D//TSC22D3//TWIST1
GO:0050678	regulation of epithelial cell proliferation	9	0.000636854	0.091473127	3.195960005	GDF5//LEP//LIMS2//RUNX3//STAT3//STAT5A//SULF1//TGFBR3//TWIST1
GO:0034097	response to cytokine	18	0.000639817	0.091473127	3.193944316	CD74//CXCL5//CXCL6//DDOST//HCK//HLA-A//HLAE//LEP//MYO1C//PF4V1//PSMB2//PTGES//PTGIS//RBMX//STAT3//STAT5A//TIMP4//TWIST1

BP, biological process.

**TABLE 2:** The top 15 enriched GO-CC terms of the methylated DEGs.

ID	Term	Count	p value	Fdr	Enrichment score	Genes
GO:0070062	extracellular exosome	28	0.000489349	0.058596438	3.310381497	AOX1//CD248//CD74//CLIC6//EFEMP1//FSTL1//HLAE//HPRT1//ISLR//KRT7//MYO1C//PGK1//PLCD1//PPL//PRELP//PSMB2//RAB5C//RBMX//RBP4//RPS9//SERPING1//SNRPD2//STEAP4//TGFBR3//THY1//VAMP8//VMO1//WWP1
GO:0043202	lysosomal lumen	5	0.000536885	0.058596438	3.27011879	CD74//CTSK//FMOD//PDGFRB//PRELP
GO:1903561	extracellular vesicle	28	0.000574475	0.058596438	3.240728952	AOX1//CD248//CD74//CLIC6//EFEMP1//FSTL1//HLAE//HPRT1//ISLR//KRT7//MYO1C//PGK1//PLCD1//PPL//PRELP//PSMB2//RAB5C//RBMX//RBP4//RPS9//SERPING1//SNRPD2//STEAP4//TGFBR3//THY1//VAMP8//VMO1//WWP1
GO:0071556	integral component of lumenal side of endoplasmic reticulum membrane	3	0.001063195	0.081334418	2.973387073	CD74//HLA-A//HLA-E
GO:0098576	lumenal side of membrane	3	0.001847225	0.107001025	2.733480203	CD74//HLA-A//HLA-E
GO:0042612	MHC class I protein complex	2	0.002098059	0.107001025	2.678182239	HLA-A//HLA-E
GO:0031901	early endosome membrane	5	0.004523861	0.188769752	2.344490779	HLA-A//HLA-E//RAB5C//STEAP4//VAMP8
GO:0030134	COPII-oated ER to Golgi transport vesicle	4	0.004935157	0.188769752	2.306699035	CD74//HLA-A//HLA-E//KLHL21
GO:0045121	membrane raft	7	0.007108918	0.191296621	2.148196479	GPM6B//HCK//MYO1C//PGK1//PTGIS//SULF1//THY1
GO:0031982	vesicle	39	0.00714269	0.191296621	2.146138182	AOX1//APH1B//C1QTNF5//CD248//CD74//CLIC6//CTSK//DDOST//EFEMP1//FSTL1//HCK//HLA-A//HLA-E//HPRT1//ISLR//KLHL21//KRT7//LHFPL2//MYO1C//PDGFRB//PGK1//PLCD1//PPL//PRELP//PSMB2//RAB5C//RBMX//RBP4//RPS9//SERPING1//SNRPD2//STEAP4//TAOK2//TGFBR3//THY1//TRAK1//VAMP8//VMO1//WWP1
GO:0062023	collagen-containing extracellular matrix	8	0.008003387	0.191296621	2.096726203	COL21A1//EFEMP1//FMOD//LAMA2//PODN//PRELP//SERPING1//SULF1
GO:0035577	azurophil granule membrane	3	0.008126981	0.191296621	2.090070775	DDOST//RAB5C//VAMP8
GO:0012507	ER to Golgi transport vesicle membrane	3	0.009313018	0.203555962	2.030909563	CD74//HLA-A//HLA-E
GO:0005689	U12-type spliceosomal complex	2	0.014088255	0.221781522	1.851142802	LSM7//SNRPD2
GO:0,045,335	phagocytic vesicle	4	0.014495524	0.221781522	1.838766071	HLA-A//HLA-E//MYO1C//VAMP8

CC, cellular component.

**TABLE 3:** The top 15 enriched GO-MF terms of the methylated DEGs.

ID	Term	Count	p value	Fdr	Enrichment score	Genes
GO:0030021	extracellular matrix structural constituent conferring compression resistance	3	0.000582628	0.178742232	3.234608341	FMOD//PODN//PRELP
GO:0042379	chemokine receptor binding	4	0.001467647	0.178742232	2.833378248	CXCL5//CXCL6//PF4V1//STAT3
GO:0008009	chemokine activity	3	0.006012581	0.178742232	2.220939072	CXCL5//CXCL6//PF4V1
GO:0000400	four-way junction DNA binding	2	0.006337918	0.178742232	2.198053411	HMGB3//RAD51C
GO:0070888	E-box binding	3	0.006722494	0.178742232	2.172469604	HDAC1//NONO//TWIST1
GO:0008201	heparin binding	5	0.009601926	0.178742232	2.017641638	CXCL6//FSTL1//PF4V1//PRELP//TGFBR3
GO:0016301	kinase activity	12	0.015562307	0.178966531	1.80792602	DYRK3//EFEMP1//HCK//NME5//PDGFRB//PDK3//PGK1//PRPS2//RIPK3//TAOK2//TGFBR3//TK1
GO:0005126	cytokine receptor binding	6	0.019817892	0.197152983	1.702942534	CXCL5//CXCL6//GDF5//PF4V1//STAT3//TGFBR3
GO:0004713	protein tyrosine kinase activity	4	0.021126016	0.197152983	1.675182406	DYRK3//EFEMP1//HCK//PDGFRB
GO:0042605	peptide antigen binding	2	0.02565908	0.207534353	1.590758917	HLA-A//HLA-E
GO:0071949	FAD binding	2	0.02565908	0.207534353	1.590758917	AOX1//STEAP4
GO:0001103	RNA polymerase II repressing transcription factor binding	2	0.028634818	0.207534353	1.543105573	HDAC1//STAT3
GO:0008200	ion channel inhibitor activity	2	0.033342478	0.21073612	1.477002122	ENSA//VAMP8
GO:0016248	channel inhibitor activity	2	0.034974515	0.21073612	1.456248298	ENSA//VAMP8
GO:0043531	ADP binding	2	0.034974515	0.21073612	1.456248298	PGK1//PRPS2

MF, molecular function.

KEGG pathway enrichment analysis showed that the top pathways related to the methylated DEGs were the chemokine signalling pathway, viral protein interaction with cytokine and cytokine receptors, the JAK-STAT signalling pathway, viral myocarditis, and the adipocytokine signalling pathway (Figures 5I,J). The top 15 enriched KEGG pathway terms of the methylated DEGs are listed in Table 4.

**TABLE 4:** The top 15 enriched KEGG pathway terms of the methylated DEGs.

ID	Term	Count	p value	Fdr	Enrichment score	Genes
hsa04062	Chemokine signaling pathway	6	0.005106388	0.52554437	2.291886204	ADCY4//CXCL5//CXCL6//HCK//PF4V1//STAT3
hsa04061	Viral protein interaction with cytokine and cytokine receptor	4	0.010010388	0.52554437	1.999549072	CXCL5//CXCL6//PF4V1//TNFRSF10D
hsa04630	JAK-STAT signaling pathway	5	0.011756369	0.52554437	1.929726788	AOX1//LEP//PDGFRB//STAT3//STAT5A
hsa05416	Viral myocarditis	3	0.014071423	0.52554437	1.851661979	HLA-A//HLA-E//LAMA2
hsa04920	Adipocytokine signaling pathway	3	0.020418909	0.52554437	1.689967473	CPT1C//LEP//STAT3
hsa01230	Biosynthesis of amino acids	3	0.025393337	0.52554437	1.595280232	PGK1//PRPS2//PYCR1
hsa05133	Pertussis	3	0.026280353	0.52554437	1.58036881	CXCL5//CXCL6//SERPING1
hsa05169	Epstein-Barr virus infection	5	0.027237069	0.52554437	1.564839633	HDAC1//HLA-A//HLA-E//RUNX3//STAT3
hsa05203	Viral carcinogenesis	5	0.027237069	0.52554437	1.564839633	HDAC1//HLA-A//HLA-E//STAT3//STAT5A
hsa04612	Antigen processing and presentation	3	0.028103977	0.52554437	1.551232219	CD74//HLA-A//HLA-E
hsa04060	Cytokine-cytokine receptor interaction	6	0.037473107	0.525800484	1.426280294	CXCL5//CXCL6//GDF5//LEP//PF4V1//TNFRSF10D
hsa05330	Allograft rejection	2	0.040811779	0.525800484	1.389214476	HLA-A//HLA-E
hsa05323	Rheumatoid arthritis	3	0.04386683	0.525800484	1.35786375	CTSK//CXCL5//CXCL6
hsa05332	Graft-versus-host disease	2	0.046843799	0.525800484	1.329347889	HLA-A//HLA-E
hsa05414	Dilated cardiomyopathy (DCM)	3	0.04745307	0.525800484	1.32373569	ADCY4//EMD//LAMA2

KEGG, kyoto encyclopedia of genes and genomes.

### ceRNA Network of circRNA-miRNA-mRNA

**FIGURE 6:**
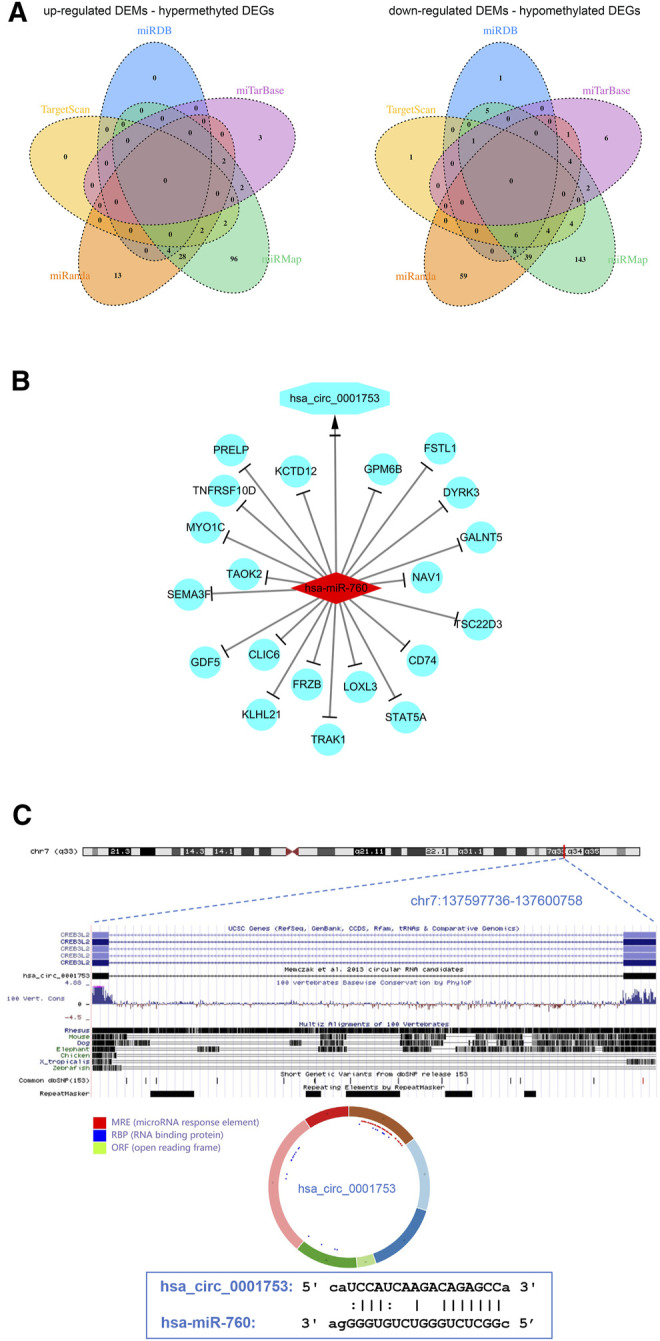
View of the circRNA-miRNA-mRNA network and circRNA structure chart. **(A)** Venn diagram of 152 upregulated DEM-hypermethylated DEG pairs and 284 downregulated DEM-hypomethylated DEG pairs. **(B)** The ceRNA network consists of 1 circRNA, 1 miRNA, and 20 mRNAs. **(C)** Has_circ_0001753 structure and hsa_circ_0001753 and hsa_miR_760 interactions identified by the CSCD database.

To better understand the interaction among the DECs, DEMs, and methylated DEGs of OS, the ceRNA network of circRNA-miRNA-mRNA was constructed. First, the miRNA binding sites of DECs were predicted by the starBase database, and a total of 7 upregulated DEC-downregulated DEM pairs and 198 downregulated DEC-upregulated DEM pairs were obtained after integration with the identified DEMs through a ceRNA mechanism. Second, the mRNA binding sites of DEMs were predicted by the miRanda, miRDB, miTarBase, TargetScan and miRMap databases, and a total of 152 upregulated DEM-hypermethylated DEG pairs and 284 downregulated DEM-hypomethylated DEG pairs were obtained after integration with the identified methylated DEGs through a ceRNA mechanism (Figure 6A). Finally, a circRNA-miRNA-mRNA regulatory network involving 1 downregulated circRNA, 1 upregulated miRNA and 20 downregulated mRNAs was visualized using Cytoscape software 3.7 (Figure 6B).

The results from the ceRNA network showed that hsa_circ_0001753 was derived from exons 12–18 of the CREB3L2 gene at chr7: 137,597,736–137,600,758 and eventually formed a mature sequence of 264 nt. The CSCD database showed that hsa_circ_0001753 contained the structure of miRNA binding sites (MREs) and RNA binding protein (RBP), suggesting that hsa_circ_0001753 is a key circRNA that potentially regulates OS development through spongy miRNA. Then, we explored the potential targeting relationship between hsa_circ_0001753 and hsa_miR_760 from the online database CSCD (Figure 6C).

### PPI Network Construction and Hub Gene Identification

**FIGURE 7:**
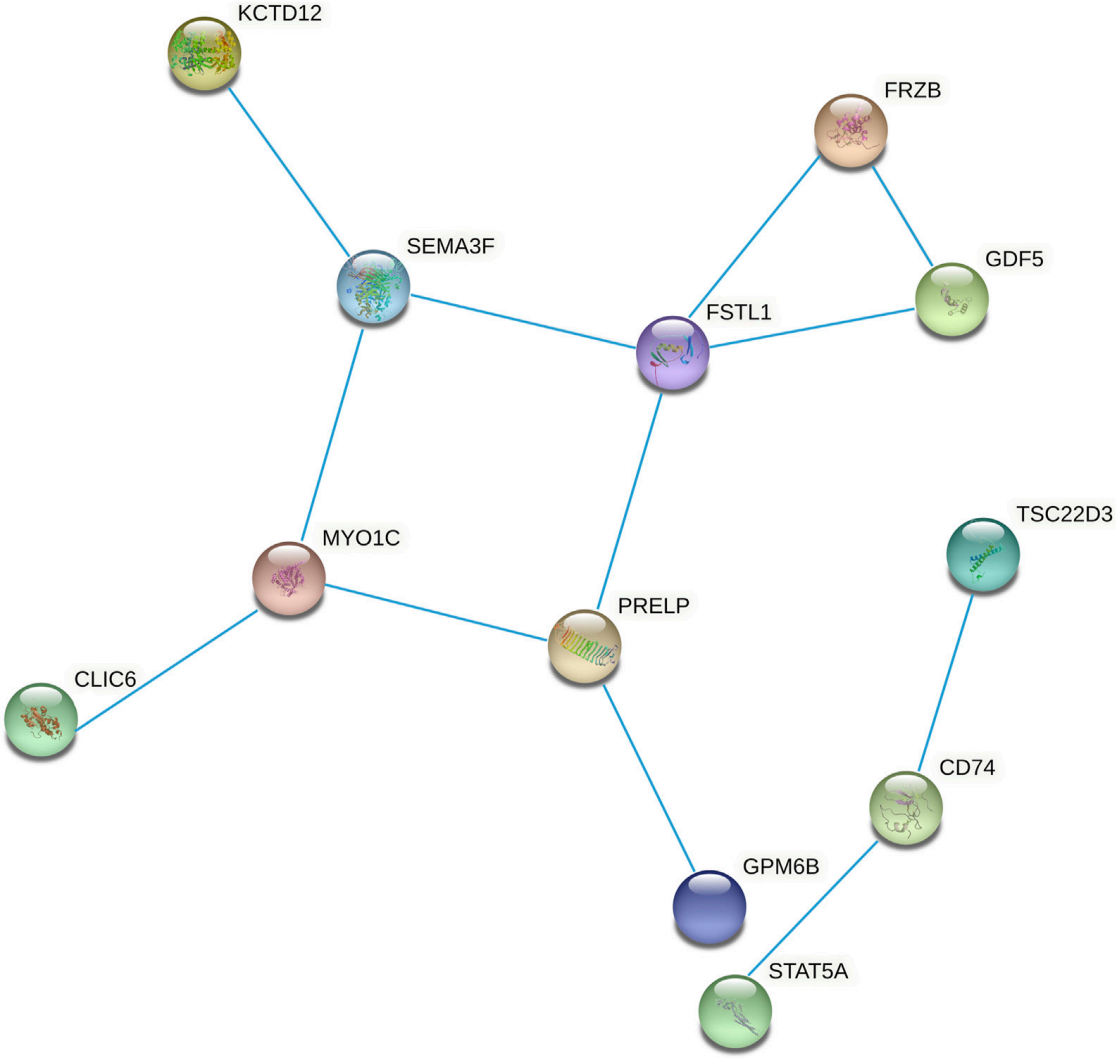
Protein-protein interaction network constructed from methylated DEGs in the ceRNA network.

We constructed the 20 mRNAs of ceRNA network expression proteins using the STRING online tool to construct PPI networks. When the mRNAs with required confidence (combined score) greater than 0.15 were submitted to STRING, a total of 12 PPI interactions were obtained, and the results are shown in Figure 7. The hub genes in the networks with a degree of connectivity greater than 2 were identified. A total of 7 hub genes were screened from the PPI network, which included CD74, FRZB, FSTL1, GDF5, MYO1C, PRELP and SEMA3F. The details of the hub genes are listed in Table 5.

**TABLE 5:** Summaries for the function of hub genes.

No.	Gene name	String ID	PPI degrees	Function
1	CD74	ENSP00000009530	2	The protein encoded by this gene associates with class II major histocompatibility complex (MHC) and is an important chaperone that regulates antigen presentation for immune response.
2	FRZB	ENSP00000295113	2	The protein encoded by this gene is a secreted protein that is involved in the regulation of bone development. Defects in this gene are a cause of female-specific osteoarthritis (OA) susceptibility.
3	FSTL1	ENSP00000295633	4	Follistatin-related protein 1; May modulate the action of some growth factors on cell proliferation and differentiation.
4	GDF5	ENSP00000363492	2	Growth factor involved in bone and cartilage formation.
5	MYO1C	ENSP00000352834	3	This gene encodes a member of the unconventional myosin protein family, which are actin-based molecular motors.
6	PRELP	ENSP00000343924	3	The protein encoded by this gene is a leucine-rich repeat protein present in connective tissue extracellular matrix. This protein has been shown to bind type I collagen to basement membranes and type II collagen to cartilage.
7	SEMA3F	ENSP00000002829	3	May play a role in cell motility and cell adhesion; Immunoglobulin like domain containing

PPI, protein-protein interaction.

### Survival Analysis of the Hub Genes

**FIGURE 8:**
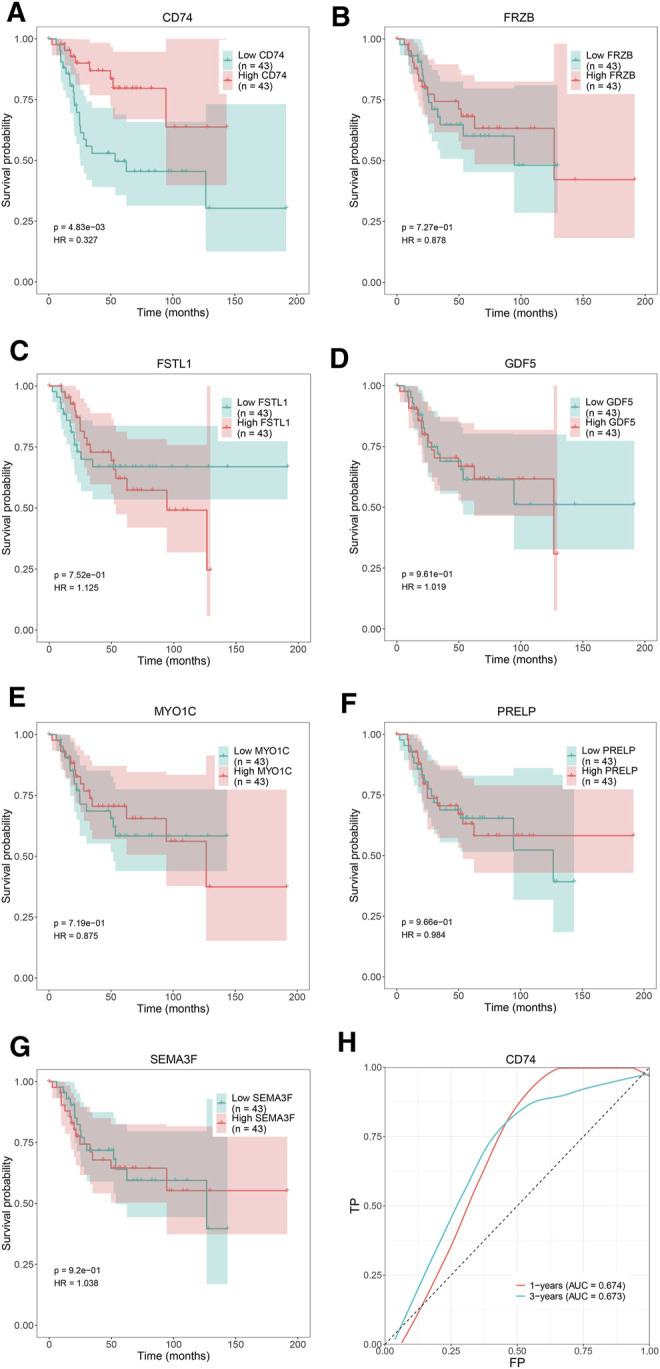
The overall survival Kaplan–Meier and ROC curves of 7 hub genes. Kaplan-Meier curves: **(A)** CD74, **(B)** FRZB, **(C)** FSTL1, **(D)** GDF5, **(E)** MYO1C, **(F)** PRELP, **(G)** SEMA3F. ROC curve: **(H)** CD74.

We attempted to investigate Kaplan-Meier curves of the 7 hub genes, and the results revealed that the overall survival time of OS patients with low expression of CD74 was lower than that of patients with high expression (*p <* 0.05; Figure 8A). However, the effects of FRZB, FSTL1, GDF5, MYO1C, PRELP, and SEMA3F expression on OS were not statistically significant (*p >* 0.05; Figures 8B–G). To determine the accurate threshold for predicting OS by CD74, an ROC curve was constructed. The ROC curve of CD74 is shown in Figure 8H, and the area under the curve (AUC) was 1 year 0.674 and 3 years 0.673, which represented good diagnostic ability for OS.

### Validation of hsa_circ_0001753/has_miR_760/CD74 in Cells and Tissues

**FIGURE 9:**
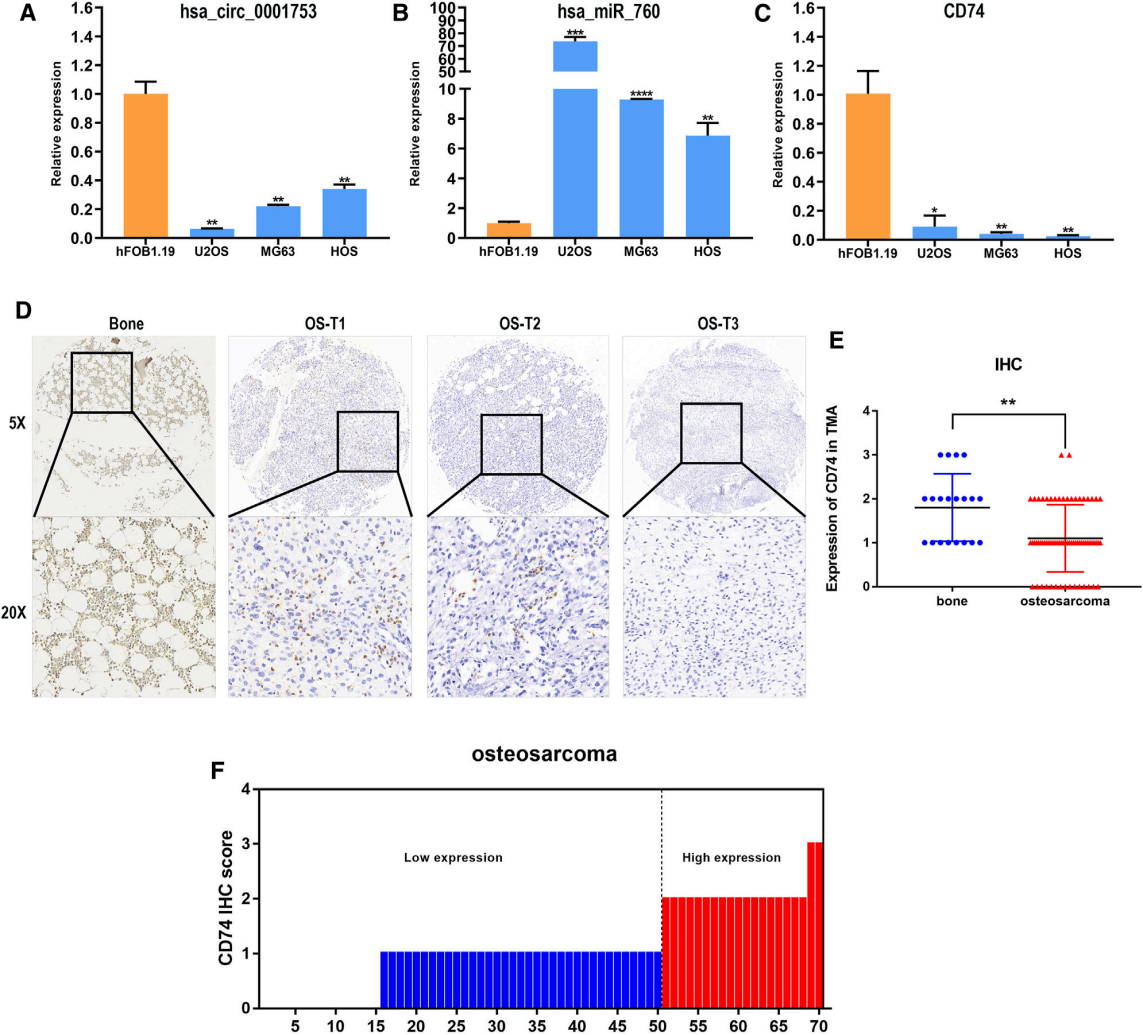
Hsa_circ_0001753/has_miR_760/CD74 expression in cell lines and tissues. **(A)** hsa_circ_0001753 was downregulated in OS cell lines compared to osteoblasts. **(B)** hsa_miR_760 was upregulated in OS cell lines compared to osteoblasts. **(C)** CD74 was downregulated in OS cell lines compared to osteoblasts. **(D)** Representative images indicated the expression of CD74 in 90 normal bone and OS tissues detected with in IHC. **(E)** Dot distribution graph of CD74 IHC staining scores was shown in 20 normal bone tissues and 70 OS tissues. **(F)** The IHC staining scores were calculated in 70 OS tissues. IHC staining score < 2 was defined as low expression, while a score ≥ 2 was regards as high expression. Data shown are mean ± SD (*n* = 3). (*p < 0.05, **p < 0.01, ***p < 0.001, ****p < 0.0001).

We constructed the hsa_circ_0001753/has_miR_760/CD74 network based on the ceRNA mechanism and the survival analysis of CD74. To test the reliability and stability of the bioinformatics analysis results, the expression patterns of this network were validated in osteoblast and OS cell lines by RT-PCR. Consistent with the bioinformatics analysis results, hsa_circ_0001753 was significantly downregulated, hsa_miR_760 was significantly upregulated and CD74 was significantly downregulated in the osteosarcoma cell lines compared to the osteoblast cell line (Figures 9A–C). This expression trend revealed that this regulatory network of hsa_circ_0001753/has_miR_760/CD74 conforms to the ceRNA mechanism. Subsequently, we utilized human tissue microarrays (TMAs) containing 70 OS samples and 20 normal bone tissue samples tissues to determine the expression of CD74 by IHC. CD74 is relatively low expressed in osteosarcoma tissue as compared to normal bone tissue (Figures 9D,E); of the 70 osteosarcoma tissue samples, 73% had low expression of CD74 (Figure 9F).

### The DNA Methylation Pattern of CD74

**FIGURE 10:**
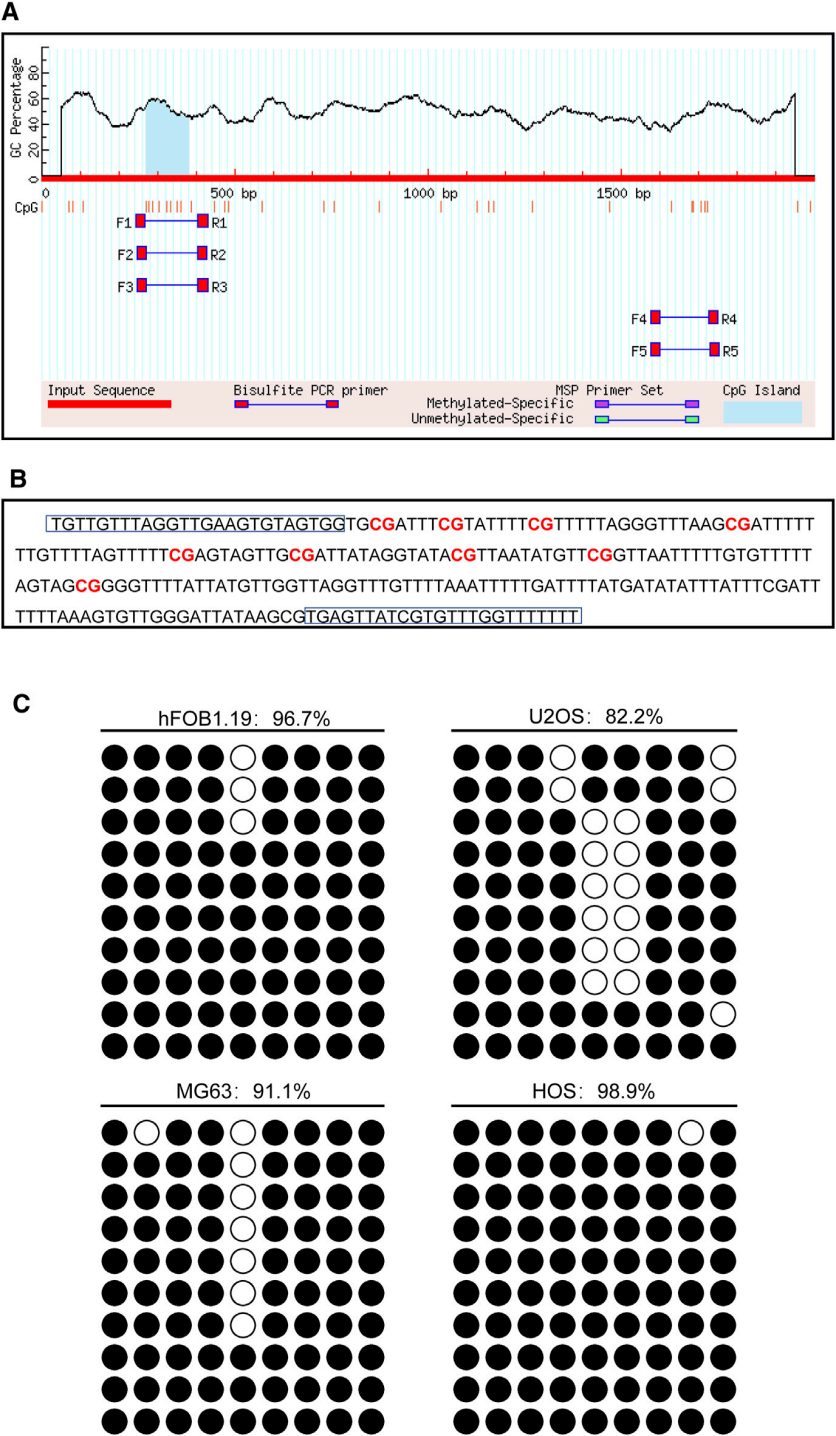
The DNA methylation pattern of CD74. **(A)** The CpG island was located between nucleotides 271 to 383 in the promoter region of CD74 gene, and there were 9 CpG sites in total. The short vertical line indicated CpG dinucleotides. **(B)** BSP primers targeting the CpG islands in the CD74 promoter region were designed and marked with red to show the CpG dinucleotides. **(C)** BSP primers targeting the CpG islands in the CD74 promoter region were designed and marked with red to show the CpG dinucleotides.

The promoter of the CD74 gene is generally considered as a 2,000 bp sequence upstream of the exon 1 site. The CpG island of the CD74 promoter was predicted using MethPrimer online software. We found that there was 1 CpG island located from 271 to 383 with 9 CpG sites in the promoter region of the CD74 gene, as shown in Figure 10A. Figure 10B shows that the BSP primer targeted the CpG island in the promoter region of the CD74 gene, and the CG dinucleotides were marked in red. DNA methylation status of the CD74 promoter were analyzed by BSP. The methylation status of the promoter region of the CD74 gene is shown in Figure 10C, and each row indicates the sequence of an individual clone. Black circles represent methylated CpG sites and white circles represent unmethylated CpG sites. The methylation rate of CD74 promoter DNA in osteoblasts was 96.7%, compared to 82.2% for U2OS, 91.1% for MG63 and 98.9% for HOS in the osteosarcoma group.

## Discussion

OS is the most common malignant bone tumour and seriously impacts the quality of life and life expectancy of patients. For osteosarcoma patients, early diagnosis and treatment of the disease is of great significance. Therefore, there is important clinical value and great market prospects for further exploring the pathogenesis of OS, finding possible therapeutic targets and realizing early diagnosis, targeted therapy and individualized treatments. Recently, microarray and high-throughput sequencing technology have been widely used in gene research on different diseases, bringing new hope for early detection, diagnosis and targeted therapy of tumours.

In our study, based on microarray datasets and using bioinformatics analysis, we identified 171 DECs, 81 DEMs, 3025 DMSs and 2,697 DEGs in the OS group compared to the control group. Using PCA, we found that the OS group was clearly separated from the control group, indicating that the screened DECs, DEMs, DMSs and DEGs were specific and could be used to identify relevant genes implicated in OS development.

To further investigate the biological pathways, we performed GSEA using the expression matrix of DEGs. The GSEA findings showed that DEGs are mainly involved in DNA replication, mitosis and cell cycle regulation signalling pathways. Genome instability is a sign of cancer, and DNA replication is the cellular process that most easily leads to cancer (Gaillard et al., 2015). Abnormal mitosis can promote the unlimited growth of defective cells, thus becoming the main method of tumour development (Mc Gee, 2015). Cell cycle disorder is the basis of abnormal cell proliferation, which is an important feature of cancer, and loss of cell cycle checkpoint control will promote genetic instability (Williams and Stoeber, 2012). Thus, the present study demonstrated that DEGs participate in vital cancer-related biological processes in OS.

GO analysis showed that the methylated DEGs were mainly enriched in terms closely related to osteosarcoma, such as cartilage development, transforming growth factor beta, signal transduction by the p53 class mediator, extracellular matrix structural constituents, extracellular exosomes and chemokine receptors. It has been reported that in addition to a large number of osteoblasts, there are also many chondrocytes formed by cartilage, indicating the important role of cartilage development in the progression of OS (Lu et al., 2014). Transforming growth factor beta can promote angiogenesis, bone remodelling, and cell migration and inhibit immune surveillance, which is beneficial to the spread and metastasis of primary tumours and plays a role in promoting tumours in OS (Verrecchia and Rédini, 2018).

p53 is a nuclear protein related to malignant transformation in several tumour model systems, and p53 gene mutations have also been found in human osteosarcoma cell lines (Masuda et al., 1987). Structural changes in the extracellular matrix (ECM) are necessary for cell migration in the process of tissue remodelling, and its components play a key role in angiogenesis and are the basis of tumour invasion and metastasis (Roomi et al., 2006). It has been confirmed in the last 10 years that exosomes are extracellular vesicles released by cells that participate in cell signal crosstalk by transferring cellular components (RNA, proteins and lipids) across cells, and can induce changes in cell behaviour, playing an important role in the progression and metastasis of OS (Chicón-Bosch and Tirado, 2020). Chemokines and their receptors have been proven to be involved in the occurrence and development of malignant tumours. The functions of specific chemokines and their receptors are closely related to the biological environment and microenvironment of their expression, and they are considered active participants in osteosarcoma biology (von Luettichau et al., 2008). Another very interesting and meaningful research result is that KEGG pathway analysis showed that the methylated DEGs were also mainly enriched in the chemokine signalling pathway. Therefore, we speculate that methylated DEGs may be involved in the development of OS by affecting extracellular matrix components, cartilage development, transforming growth factors, exosomes, chemokine receptors, and signal transduction pathways of p53 mediators and may be a potential target for diagnosis and treatment.

Based on the ceRNA mechanism and methylated DEGs in OS, we constructed a circRNA-miRNA-mRNA regulatory network and identified the hub genes. The overall survival of CD74 with low expression levels in the 7 hub genes was shorter than that with high expression levels, while the area under the curve (AUC) of CD74 represented a good diagnostic capability for OS. We also validated the expression pattern of hsa_circ_0001753/has_miR_760/CD74 in osteoblasts and OS cell lines, thus confirming that the ceRNA regulatory molecular network hsa_circ_0001753/has_miR_760/CD74 potentially plays important roles in the pathogenesis of OS. Compared with normal bone TMAs, the expression of CD74 in OS TMAs is lower. CD74 was hypomethylated in osteoblast and hypermethylated in osteosarcoma cells as compared to DNA methylation patterns in 19 osteosarcoma cell lines and 2 normal osteoblasts in the GSE36002 datasets. These results fully demonstrated that the expression level of hypermethylation-modified CD74 gene was decreased. Unfortunately, analysis of DNA methylation patterns in osteoblast and osteosarcoma cells using BSP did not reveal significantly different trend results. We suggested that this might be due to the fact that the type and number of osteoblastic and osteosarcoma cell lines we used were different from the GSE36002 datasets, so that no clear trend of difference was observed in our verification results. CD74, also known as the MHC class II-associated invariant chain, is a transmembrane glycoprotein that plays a vital role as a chaperone of MHC class II proteins during antigen presentation. CD74 is a cell surface receptor for macrophage migration inhibitory factor and is associated with tumour progression and metastasis. Its expression can be used as a prognostic factor for many cancers, and its high relative expression can be used as a marker of tumour progression (Gil-Yarom et al., 2017). Studies have shown that the overexpression of CD74 in thyroid cancer is associated with advanced tumour staging and can be used as a therapeutic target. Treatment of thyroid cancer cells with anti-CD74 antibodies inhibits cell growth, colony formation, cell migration and invasion, and secretion of vascular endothelial growth factors (Cheng et al., 2015). Another study revealed the mechanism of CD74 in the proliferation, invasion and angiogenesis of bladder cancer; indicating that it can be used as a potential therapeutic target for bladder cancer (Gai et al., 2018). Of interest, CD74 is upregulated in various forms of cancer and is associated with proliferative and metastatic potential but is downregulated in several cancers, including OS. High expression of CD74 in brain metastatic tumour cells can cause the processing of functional HLA class II cells, and is associated with a better prognosis (Zeiner et al., 2018). Another study confirmed that CD74 is a useful tumour cell prognostic protein marker closely related to recurrence-free survival and overall survival in stage III melanoma (Ekmekcioglu et al., 2016). We speculate that CD74 may also participate in the occurrence and development of OS by regulating malignant biological behaviours such as cell proliferation, migration, and invasion.

Before our study, there were also some related studies on the circRNA of OS. A study by Liu et al. (2017) on microarray expression profile and functional analysis of circRNA in osteosarcoma provided a good basis for our study. In their study, we first reported the comprehensive expression profile of circRNA in osteosarcoma and constructed a ceRNAs prediction network. However, on the basis of this study (GSE96964), we have performed a more in-depth analysis by combining miRNA (GSE70367), mRNA (GSE42351 and GSE33382) and DNA methylation data (GSE36002) from other osteosarcoma samples and constructed a new regulatory network of ceRNA. The large number of samples in multiple databases may provide more reliable research results. In addition, we verified the reliability of the regulatory network on cell lines, and conducted immunohistochemical verification on a large number of sample tissues. The hsa_circ_0001753/has_miR_760/CD74 network constructed based on competing endogenous rna mechanisms and dna methodology may serve as a target for the early diagnosis and specific treatment of OS, and the findings can provide novel insights into the pathogenesis of OS.

Although rigorous bioinformatics analysis was adopted in this research and some results were achieved, it still has some limitations. Due to the small amount of cell sample used to detect the methylation pattern of CD74, and OS tumours are highly heterogeneous, our results need to be further verified with clinical samples to obtain more accurate results. To identify the potential molecular mechanism of OS, additional *in vivo*/*in vitro* experimental studies are necessary.

## Conclusion

In summary, this study identified DECs, DEMs, DMSs and DEGs related to OS. Methylated DEGs were also mainly concentrated in pathways related to osteosarcoma. On the basis of the ceRNA mechanism and methylated DEGs, the hsa_circ_0001753/has_miR_760/CD74 network was constructed and validated in cell lines and tissues. Low expression of CD74 is associated with poor overall survival time and presents a good diagnostic ability. Methylated DEGs may be involved in the occurrence and development of OS, and hsa_circ_0001753/has_miR_760/CD74 may be a target for early diagnosis of and specific treatment for OS. Using integrated bioinformatics analysis of integral competing endogenous RNA mechanisms and DNA methylation may provide valuable information for exploring the potential pathogenesis of OS.

## Data Availability

The datasets presented in this study can be found in online repositories. The names of the repository/repositories and accession number(s) can be found in the article/Supplementary Material.
